# V-Gel^®^ Guided Endotracheal Intubation in Rabbits

**DOI:** 10.3389/fvets.2021.684624

**Published:** 2021-08-10

**Authors:** Alessandra Fusco, Hope Douglas, Adriana Barba, Klaus Hopster, Darko Stefanovski, Benjamin Sinder, Patrick J. Cahill, Brian Snyder, Thomas P. Schaer

**Affiliations:** ^1^Department of Clinical Studies New Bolton Center, School of Veterinary Medicine, University of Pennsylvania, Kennett Square, PA, United States; ^2^Division of Orthopaedics, Children's Hospital of Philadelphia, Philadelphia, PA, United States; ^3^Department of Orthopaedic Surgery, Children's Hospital, Harvard Medical School, Boston, MA, United States

**Keywords:** rabbit, anesthesia, intubation, endotracheal tube, blood gas, endoscopy

## Abstract

**Background:** General anesthesia in rabbits is associated with higher morbidity and mortality relative to other mammalian species commonly anesthetized. Unique challenges related to endotracheal intubation (ETI) in rabbits contribute to this risk.

**Objective:** To improve the safety of ETI in rabbits, we developed two new ETI methods using a supraglottic airway device (v-gel^®^) to facilitate ETI and compared them to traditional “blind” technique. We hypothesized that relative to blind ETI, v-gel^®^ guided ETI provides more successful placement of the endotracheal tube (ETT) in a shorter time. Outcomes included number of intubation attempts, time for achievement of ETI, endoscopic findings, and serial arterial blood gas (ABG) analysis.

**Study Design:** Prospective, randomized, and crossover study.

**Methods:** Ten female, New Zealand White rabbits aged 1–2 years old, weighing 4.3 ± 0.4 kg, were anesthetized four times. Each time, ETI was performed with one of the following techniques: Method 1: v-gel^®^ guided, polypropylene catheter facilitated, intubation using a cuffed ETT; Method 2: v-gel^®^ guided intubation using an uncuffed ETT directly inserted through the device airway channel; Method 3 and 4: Blind intubation with uncuffed or cuffed ETT. Upper airway endoscopy was performed before intubation attempts and after extubation. Serial ABG analysis was performed during the peri-intubation process.

**Results:** V-gel^®^ guided techniques allowed successful ETI on the initial attempt for 9/10 subjects using Method 1 and 10/10 using Method 2. Relative to the v-gel^®^ guided techniques, the blind techniques required more intubation attempts. A median of 2 attempts (range 1–4, *p* < 0.007) were required for the uncuffed ETT, and a median of 4 (range 1–4, *p* < 0.001) attempts were performed for the cuffed ETT. The time to perform successful ETI was positively correlated with the number of attempts (ρ = 0.82), while successful ETI was negatively correlated with number of attempts (ρ = −0.82). Endoscopic findings showed mild to moderate laryngeal trauma. In the absence of oxygen supplementation, ABG analysis demonstrated low PaO_2_, while PaCO_2_ remained consistent.

**Conclusions:** Facilitated ETI using the v-gel^®^ guided techniques allows for the rapid establishment of a secure airway to provide ventilatory support for rabbits undergoing general anesthesia.

## Introduction

Rabbits are used extensively in biomedical research and enjoy great popularity as companion animals. Rabbits commonly undergo general anesthesia during *in vivo* research investigations and as veterinary patients. Anesthesia and sedation in rabbits carry a higher risk relative to other laboratory and companion animal species with a 1.39% incidence of anesthetic-related deaths reported. This is 6–8 times higher than in dogs and cats (1). Species related factors that contribute to this risk include a narrow therapeutic range of anesthetic agents, a recognized sensitivity to the gastrointestinal and cardiorespiratory effects and stresses of general anesthesia, and higher tendencies for pronounced hypothermia and hypoventilation (1). In addition, underlying respiratory diseases, such as *Pasteurella multocida* infections, have been documented, which could increase the risk of respiratory complications (1, 2). Due to increased respiratory complications, rabbits undergoing procedures under general anesthesia benefit from endotracheal intubation (ETI) to provide ventilatory support and airway security. However, ETI in rabbits is technically challenging and more difficult than in other commonly anesthetized veterinary species. Direct visualization of the larynx in rabbits is difficult due to their specific anatomical features such as a deep and narrow oropharynx with limited mandibular excursion, a relatively large tongue, and large incisors (2–4). In addition, the diameter of the laryngeal opening is smaller when compared to that of the trachea, and laryngeal stimulation can cause severe laryngospasm and laryngeal trauma (5). Therefore, ETI requires experience and an effective technique.

Due to the challenges of ETI in rabbits, several techniques have been explored and are employed based on individual preference, clinical presentation, and equipment availability. A blind orotracheal intubation technique is commonly employed, which involves extension of the head and neck and passage of the orotracheal tube through the larynx and into the trachea. This traditional technique requires little additional equipment aside from the endotracheal tube (ETT) itself. However, this technique is technically challenging as it requires experience in the tactile sensations involved in ETT passage through the opening of the glottis and the detection of changes in breathing sounds (6). In addition, multiple attempts may be required to successfully intubate the animal, and thus the intubation process can be prolonged (7, 8). Alternatives to the traditional blind approach have been described, including endoscopic-guided intubation (9–11), direct visualization through laryngoscopy (12), nasotracheal intubation (13), retrograde orotracheal intubation (14), tracheostomy (15), capnography guided intubation (16), and intubation supported by esophageal cannulization (17). Another alternative method that has been proposed is intubation using a polypropylene catheter as a guide for insertion of the ETT (5). In this technique, direct laryngoscopy is used to view the laryngeal opening, which may not be feasible in very young rabbits or smaller breeds. Due to the challenges with ETI, a supraglottic airway device (v-gel^®^) has been developed for use in rabbits (18). This device has documented ability for provision of airway management and ventilatory support (8). However, some disadvantages, such as laryngeal compression and lingual cyanosis, have been reported together with an increased risk of gastric tympanism and a greater gas leakage during controlled mechanical ventilation (CMV) compared with an ETT (19). In addition, the v-gel^®^ is sensitive to movement and can be shifted out of place with positional changes of the patient (11). The overall objective of this prospective, randomized, non-blinded, cross-over study was to improve the safety of ETI in rabbits. We developed two new ETI methods using a supraglottic airway device, v-gel^®^, and compared them with the traditional “blind” ETI technique. We hypothesized that the v-gel^®^ guided ETI techniques would have a higher success rate and be completed in a shorter amount of time when compared to the traditional blind technique. To further compare the two new methods relative to the traditional ETI, endoscopy, and arterial blood gas analysis were also performed at different stages of the intubation process.

## Materials and Methods

### Animals

This study was reviewed and approved by the Institutional Animals Care and Use Committee of University of Pennsylvania. Ten female, skeletally mature, New Zealand White rabbits aged 1–2 years and weighing 3.5–5.1 kg (4.3 ± 0.4), were enrolled in the study. The animals were internally sourced, and the study group included does that were delivered from a commercial supplier (Charles River Laboratory Inc.) with kits that were designated for another study. Additionally, kits that had become skeletally mature and did not participate in that separate study were included in this investigation. The rabbits were housed in stainless steel cages with auditory, olfactory, and visual contact. Their diet consisted of a pelleted diet and a small amount of fresh roughage, with free access to hay and water. Animals were housed in a room with a 12:12 h light cycle and controlled temperature (70.82 ± 0.1°F) and humidity (35.9 ± 6.2%). All animals were monitored daily by a veterinarian from the principal investigator's laboratory.

### Anesthesia

Each rabbit underwent a total of four anesthetic events, with a 7-day washout period between each experiment. The sequence of rabbits undergoing the experiment was determined *a priori* by randomly attributing a number from 1 to 10 to each rabbit ear tag. The sequence of different intubation methods was then randomized for each rabbit. The randomization was performed using Microsoft Excel (2019) by a non-study affiliated individual and allocation was not concealed. An *a priori* performed power analysis using an alpha error of 0.05 and a beta error of 0.8 revealed that 10 rabbits would be necessary per group to detect clinically relevant differences between groups with regards to intubation attempts.

Rabbits were induced with an intramuscular injection of ketamine (35 mg/kg) (Ketamine HCl, Covetrus) and xylazine (2.5 mg/kg) (Xylazine Injection, Covetrus). Propofol (PropoFlo^TM^ 28, Zoetis) was administered as intravenous bolus (0.5–1 mg/kg) if anesthetic depth was not sufficient to perform the intubation procedure. The following metrics were evaluated to determine sufficient anesthetic depth for intubation: jaw tone, protraction of the tongue, presence of swallowing or cough in response to the insertion of the v-gel^®^ or ETT, purposeful movement, presence of withdrawal reflex, and chewing.

Glycopyrrolate (0.01 mg/kg) (Glycopyrrolate injection, BluePoint) was administered intravenously if bradycardia (defined as heart rate <120 beats per minute) with concurrent hypotension (defined as mean arterial pressure <60 mmHg) occurred. Atipamezole (0.1 mg/kg) (Antisedan, Zoetis) was administered intramuscularly at the end of the experimental procedure.

### Intubation Methods and Monitoring

**Figure 1:**
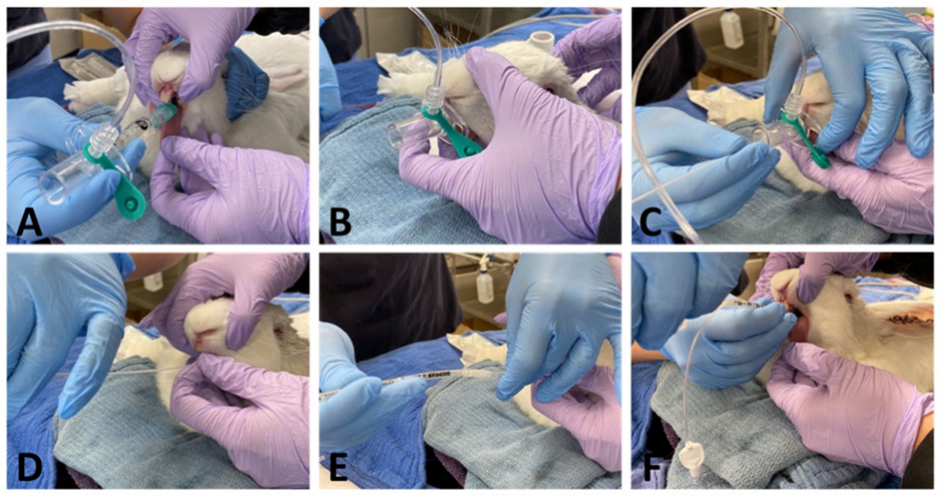
Representative pictures showing ETI performed with method 1. A size R4 v-gel^®^ is inserted **(A)** and correct placement is verified by capnography **(B)**. A 3.5 Fr polypropylene guide catheter is then inserted through the v-gel^®^ airway channel **(C)**. The v-gel^®^ is then removed and a ETT is threaded over the guide, with the head of the rabbit kept in a neutral position, without extension of the neck **(D-F)**.

**Figure 2:**
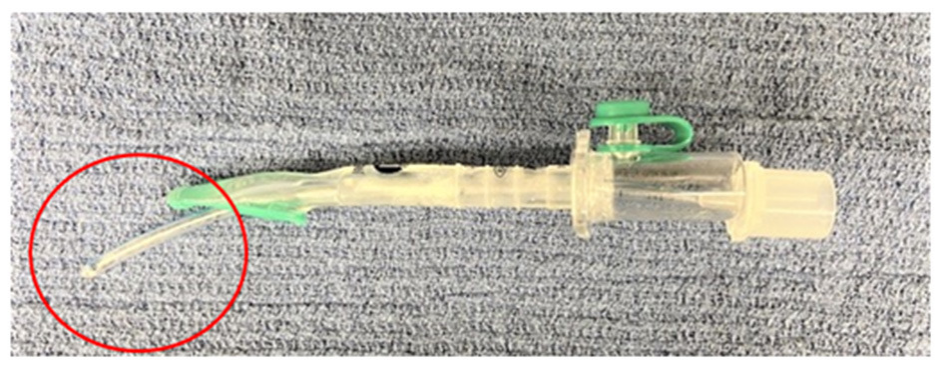
Representative picture showing a 2.5 mm internal diameter uncuffed ETT inserted through the v-gel^®^ airway channel (note the endotracheal portion of the tube circled in red).

Approximately 10 min after premedication, the right or left auricular vein and artery of the same ear (alternating ears between experimental events) were catheterized with a 24 Ga (artery) and a 22 Ga catheter (vein) (Surflo, Terumo), respectively. The distance between the incisors and the thoracic inlet was measured and recorded for each animal. For each anesthetic session, after the pre-intubation endoscopy, and at least a period of 5 min of pre-oxygenation, ETI was performed with one of the following modalities in a randomized order:

*Method 1*: Figure 1 after placement of the v-gel^®^ (v-gel^®^, Docsinnovent), confirmed by capnography (Figures 1A,B), a lubricated 3.5 French (~1.2 mm external diameter), 40 cm polypropylene catheter (Sovereign Urinary Catheter, Henry Schein Animal Health) was inserted through the v-gel^®^ airway channel (Figure 1C). The v-gel^®^ was then removed and a 3 mm internal diameter (ID) cuffed ETT (Sheridan/CF^®^, Teleflex Medical) was threaded over the catheter (Figures 1E,F). After insertion of the ETT, the polypropylene catheter was removed.
*Method 2*: After v-gel^®^ placement, a 2.5 mm ID uncuffed ETT (Sheridan Uncuffed, Teleflex Medical) was inserted directly into the v-gel^®^ airway channel and both devices were left in place with the endotracheal portion of the tube coming through the v-gel^®^ laryngeal seal (Figure 2).
*Method 3*: Blind intubation with an uncuffed 3 mm ID ETT (Sheridan Uncuffed, Teleflex Medical).
*Method 4*: Blind intubation with a cuffed 3 mm ID ETT (Sheridan/CF^®^, Teleflex Medical).

A size R4 v-gel^®^ was used for all the rabbits. The size of ETT and v-gel^®^ were chosen based on rabbits' average size and weight. The ETT used in *Method 2* was smaller and uncuffed because of the limited diameter of the R4 v-gel^®^ airway channel.

Pulse oximetry, ECG, invasive blood pressure (IBP), FiO_2_, end-tidal carbon dioxide (ETCO_2_), and respiratory rate (RR) were recorded throughout each anesthetic event using a multiparameter monitor (Datex Ohmeda S/5, GE). Rectal temperatures were recorded throughout the anesthetic session, except during intubation. The rabbits were maintained in sternal recumbency with the forelimbs and hind limbs extended and head aligned with the spine. Topical 2% lidocaine (0.2 ml) (Lidocaine 2%, VetOne) was applied via atomizer to the larynx 2 min prior to intubation. The v-gel^®^ was lubricated with lidocaine gel (Lidocaine Hydrochloride Oral Topical Solution 2%, Akorn) mixed with regular sterile lubricant (VetOne^®^ OB Lube), and the ETT was lubricated (VetOne^®^ OB Lube). Intubation was performed with one of the above-mentioned modalities by the same investigator (AF). This experimenter was selected based on her experience and skills with the traditional rabbit intubation technique, having taken part to numerous experiments as a veterinarian and researcher. A second person (HD), with extensive experience in small animal and rabbit anesthetic procedures, gently extended the tongue to the left side of the mouth and helped maintain the rabbit's head in optimum position for each experiment.

For *Methods 1* and *2*, the rabbit's head was extended gently to facilitate v-gel^®^ placement. Correct placement of the v-gel^®^ was confirmed via capnography. The animal's head was then returned to a neutral position (Figures 1C–E) and the polypropylene guide or the ETT inserted into the v-gel^®^ airway channel. For both blind techniques, the neck was maintained in extension. The head was maintained in extension during blind intubation attempts.

The number of intubation attempts were recorded along with the total time for ETT insertion (time from the start of the first intubation attempt until successful intubation with capnographic confirmation). After four unsuccessful attempts, the intubation procedure was considered a failure. For each attempt, the ETT was completely inserted and connected to the capnography. An attempt was considered failed if no waveform could be observed on the capnogram and no end-tidal carbon dioxide was registered. Supplemental oxygen (O_2_) was provided via ETT or via mask when ETI failed throughout the recovery period. After intubation, the animals were connected to a circle breathing system designed for animals under 7 kg body weight (Anesthesia WorkStation, Hallowell EMC). This system features a ventilator that utilizes a floating puck rather than a bellows to separate the ventilator drive gas from the patient breathing circuit (20). This system allows both for spontaneous and controlled mechanical ventilation. Once connected to the circuit, the rabbits breathed spontaneously on 100% oxygen at 1L/min for at least 10 min. Fraction of inspired oxygen (FiO_2_), respiratory rate (RR) and end tidal concentration of CO_2_ (ET CO_2_) were recorded every 5 min. FiO_2_ and end tidal CO_2_ were measured with sidestream capnography and gas analysis. For all animals intubated with cuffed ETT, the cuff was inflated to 20 mmHg (19) measured with an aneroid manometer connected via a three-way stopcock to the cuff balloon. Once the intubated animals were connected to the breathing system, the system leak was measured and recorded. After connection to the breathing circuit, the oxygen fresh gas flow was adjusted from 1 L/min until the flow at which point the floating puck did not return to its original position at the end of expiration. This level was then considered the total system leak.

### Upper Airway Endoscopy

**Figure 3:**
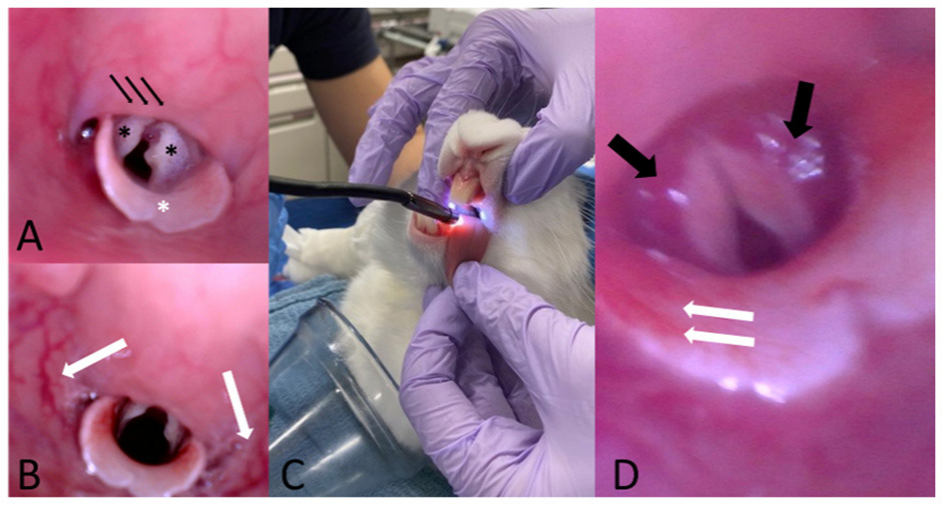
**(A)** Representative pre-intubation image of normal endoscopic appearance of the laryngeal structures in one of the study animals. Note the two arytenoids (black asterisks), the epiglottis (white asterisk) and the soft palate (black arrows). **(B)** Representative post-intubation endoscopic image showing hyperemia of the pharyngeal walls. **(C)** Representative picture showing the scope insertion into the oral cavity with the rabbit maintained in sternal recumbency. One assistant gently pulls the tongue to facilitate the passage of the scope and head and neck are maintained extended. **(D)** Representative post-intubation endoscopic image showing bilateral hyperemia of the arytenoids (black arrows) and hyperemia of the epiglottis (white arrows).

Endoscopy of the upper airway was performed before intubation and after extubation (or after failed ETI) for macroscopic evaluation of the laryngeal structures (Figure 3) with a dual lens rigid endoscope, 70°, 8 mm diameter (Teslong Dual Lens Endoscope Camera, 1080P). Pre- and post-intubation endoscopic images were obtained to evaluate the laryngeal structures for the presence of gross macroscopic abnormalities (edema, hyperemia, hemorrhage, traumatic mucosal lesions, and presence of mucus).

### Arterial Blood Gas Analysis

Arterial blood samples were collected at 4 different time points. T_0_: 10–15 min after premedication (pre O_2_ administration), T_1_: immediately before intubation (after at least 5 min of pre-oxygenation), T_2_: immediately after intubation attempts before provision of O_2_ from the anesthetic breathing circuit, and T_3_: 10 min after completion of the intubation procedure (after provision of O_2_ via ETT or mask if ETI failed) and analyzed with a blood gas and electrolyte analyzer (Opti CCA TS2, OPTIMedical).

### Anesthetic Recovery

After extubation, rabbits were provided oxygen supplementation with a mask until responsive to stimulation with pedal reflex or showing chewing movements. Oxygen supplementation was then terminated and SpO_2_ was continuously monitored with portable pulse oximetry. HR, RR, and rectal temperature were monitored at regular intervals of 5–10 min until the rabbit resumed spontaneous movements. Presence of blood staining on the tube and complications such as edema, bruising, or cyanosis of the tongue were also recorded.

### Statistical Analysis

Statistical analysis was performed on the intubation procedure data and the ABG data. All analyses were conducted in Stata (Stata 16.1MP, StataCorp), with two-sided tests of the hypotheses and a *p* < 0.05 as the criterion for statistical significance. Descriptive analyses include computation of means standard deviations for normally distributed data and medians and interquartile ranges (IQR) for not-normally distributed continuous variables. Tests of normal distribution were performed to determine extent of skewness. Frequency counts and percentages were used for categorical variables. For the intubation data, univariate analysis was conducting using Spearman's rank correlation test in pairwise fashion to compare number of attempts with success and the duration of the intubation procedure. The multivariable model included the type of intubation, anesthetic event, and weight as fixed effects. Duration of intubation was analyzed using a mixed-effects Poisson regression model. The type of intubation and interaction between anesthetic event and weight were considered as fixed effects in the model. Random effects were set on the level of the individual animal. Mixed effects ordered logistic regression was used to analyze the outcome and the number of attempts for successful intubation. The model included type of intubation, anesthetic event, and weight of animals as fixed effects. Random effects were set on the level of individual animal. The arterial blood gas data were analyzed using Bayesian mixed-effects linear regression. Time and type of intubation were considered as fixed effects in the multivariable model for arterial blood gas analysis.

## Results

The investigator who performed the intubations was consistent throughout the course of the study, this investigator could not be blinded, and it is possible that experiences gained throughout the course of the study could have impacted the results. However, the effect of this experience was attempted to be reduced by performing the trials in a randomized order.

All rabbits uneventfully recovered from each anesthetic event and did not experience any complications during the study. A full data set was collected from most of the animals. ETCO_2_ values were not recorded in one animal intubated with cuffed ETT. Complete separation or perfect prediction of the datasets was not possible. This can occur when a subgroup of animals does not exhibit variability in terms of the outcome leading to problems with the estimation of likelihood, inference statistical analysis of the binary variable of successful intubation, therefore a Firth's penalized logistic regression was applied.

To achieve suitable anesthesia depth for trial completion, propofol administration (PropoFlo^TM^ 28, Zoetis) as 0.5–1 mg/kg boluses was necessary in eight animals. In six of them, only one bolus was administered over the course of a single anesthetic event after extubation to permit endoscopy and final data acquisition. In the remaining two animals, multiple propofol boluses were administered over the course of the procedure, in three of the four anesthetic events, to maintain consistent experimental operating conditions. A single IV bolus of glycopyrrolate (0.01 mg/kg) was administered to eight animals to treat bradycardia (HR <120) with concurrent hypotension (mean ABP <60 mmHg) before or after the intubation procedure. In three of the trials, one rabbit required glycopyrrolate shortly after premedication, before ETI was performed. During two of the trials, two animals received glycopyrrolate, one before and one after ETI. The other five rabbits received glycopyrrolate for only one of the trials, two before ETI and three after ETI. The distance measured between incisors and thoracic inlet was between 13 and 15 cm.

**Figure 4:**
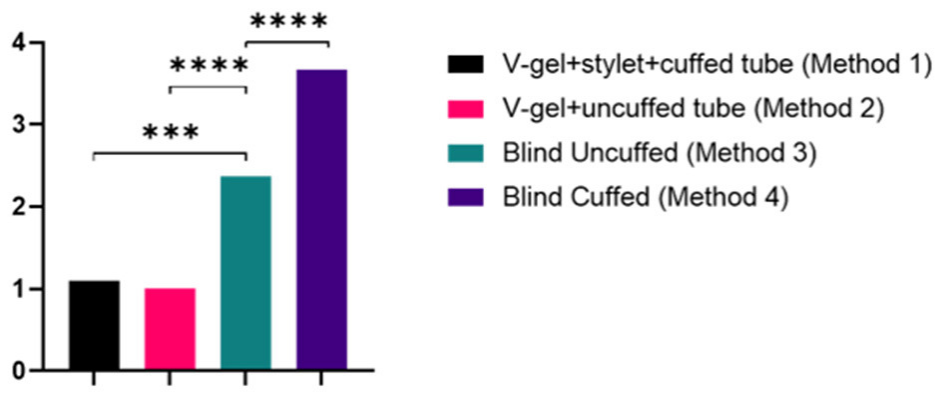
Graph of the number of attempts for each intubation method. Data are presented as mean and statistically significant differences are indicated by lines and asterisks.

ETI using both v-gel^®^ guided techniques (*Methods 1* and *2*) required significantly fewer attempts to achieve a secure airway compared to blind ETI (*p* < 0.007 for uncuffed ETT; *p* < 0.001 for cuffed ETT; Table 1 and Figure 4). ETI was successful on the initial attempt using the v-gel^®^ guided intubation for 90% (*n* = 9/10) of animals intubated with *Method 1*. Suspected laryngospasm interfered with the initial ETI attempt in one rabbit, remedied by administering an IV bolus of propofol to achieve success on the second attempt. Intubation was achieved on the initial attempt in 100% (*n* = 10/10) of animals intubated with *Method 2*. Blind ETI using an uncuffed ET (*Method 3*) was 80% (*n* = 8/10) successful in achieving a secure airway, often requiring two or more attempts (median = 2). Blind intubation with cuffed ETT (*Method 4*) often required four attempts (median 4) and was successfully accomplished in only one rabbit. The number of intubation attempts was positively correlated with ETI procedure duration (ρ = 0.82; *p* < 0.001). The total time for achieving intubation was 2.4 ± 0.9 min for *Method 1*, 1 ± 0.3 min for *Method 2*, 3.1 ± 2.3 min for *Method 3*, and 4.8 ± 1.8 min for *Method 4* (Table 1).

**Table 1:** Intubation data.

**Intubation modality**	**Number of attempts**	**Duration (min)**	**% success**
*Method 1* (v-gel^®^ + Stylet + cuffed ET)	1–2 (1)	1.4–4.4 (2.4 ± 0.9)	100 (*n =* 10/10)
*Method 2* (v-gel^®^ + Uncuffed ET)	1 (1)	0.6–1.6 (1 ± 0.3)	100 (*n =* 10/10)
*Method 3* (Blind Uncuffed)	1–4 (2)	0.7–6.5 (3.1 ± 2.3)	80 (*n = 8*/10)
*Method 4* (Blind Cuffed)	1–4 (4)	0.7–6.4 (4.8 ± 1.8)	10 (*n =* 1/10)

*Number of attempts, duration in minutes, and percentage of success. Data are presented as range and median (number of attempts) or mean and standard deviation (duration)*.

**Figure 5:**
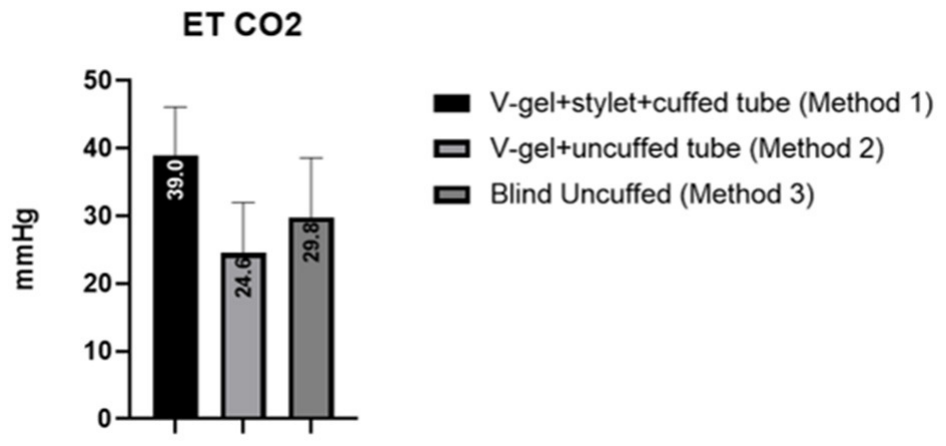
Graph displaying the ETCO_2_ values measured for the different intubation techniques. Values are reported as mean (within columns) and standard deviation.

The mean and standard deviation (SD) of RR, HR, and SpO_2_ values recorded pre-, during, and post-intubation are reported in Table 2. The baseline leak of the anesthesia machine alone was 200 ml/min. In general, the air leak was higher for rabbits intubated with an uncuffed ETT (445 ± 59.9 ml/min) compared to a cuffed ETT (300 ± 100 ml/min), independent of the method used to achieve ETI (v-gel^®^ guided vs. blind). In rabbits intubated with *Method 2*, the leak measured was higher when the v-gel^®^ and the ETT were both in place compared to v-gel^®^ alone (366.7 ± 81.6). The effect of the air leak was reflected in the mean ETCO_2_ (Figure 5), which was higher (39 ± 7 mmHg) in rabbits with cuffed ETT (*Method 1*) compared to rabbits with uncuffed ETT (24 ± 7 mmHg for *Method 2*; 30 ± 9 for *Method 3*). ETCO_2_ values were not recorded for the one animal that was successfully intubated with *Method 4*.

**Table 2:** Respiratory rate, heart rate, and SpO_2_ values recorded pre-, during, and post-intubation.

**Intubation modality**	**Pre-intubation**	**Intubation**	**Post-intubation**
Respiratory rate (RR, breaths/min)	35.3 ± 12.2	35.6 ± 10.4	36.7 ± 13.3
Heart Rate (HR, beats/min)	168.6 ± 18.9	177.4 ± 20.1	170.8 ± 21.1
Pulse oximetry (SpO_2_)	97.5 ± 4.3	96.4 ± 4.4	98.2 ± 2.8

A total of 50 endoscopic upper airway images were of sufficient quality to allow macroscopic assessment of the larynx and glottis. Across all cohorts, hyperemia was the most common finding before and after intubation, followed by edema and hemorrhage, with severities ranging from mild to moderate. Mucosal trauma and the presence of mucus were occasionally detected. Over the course of the experiments, trace blood staining of the exterior of the ETT following extubation was noted for one rabbit (*Method 1*). No clinical signs of significant upper or lower airway trauma (lingual cyanosis, lingual edema, coughing, and respiratory distress) were observed in any of the animals in this study.

**Figure 6:**
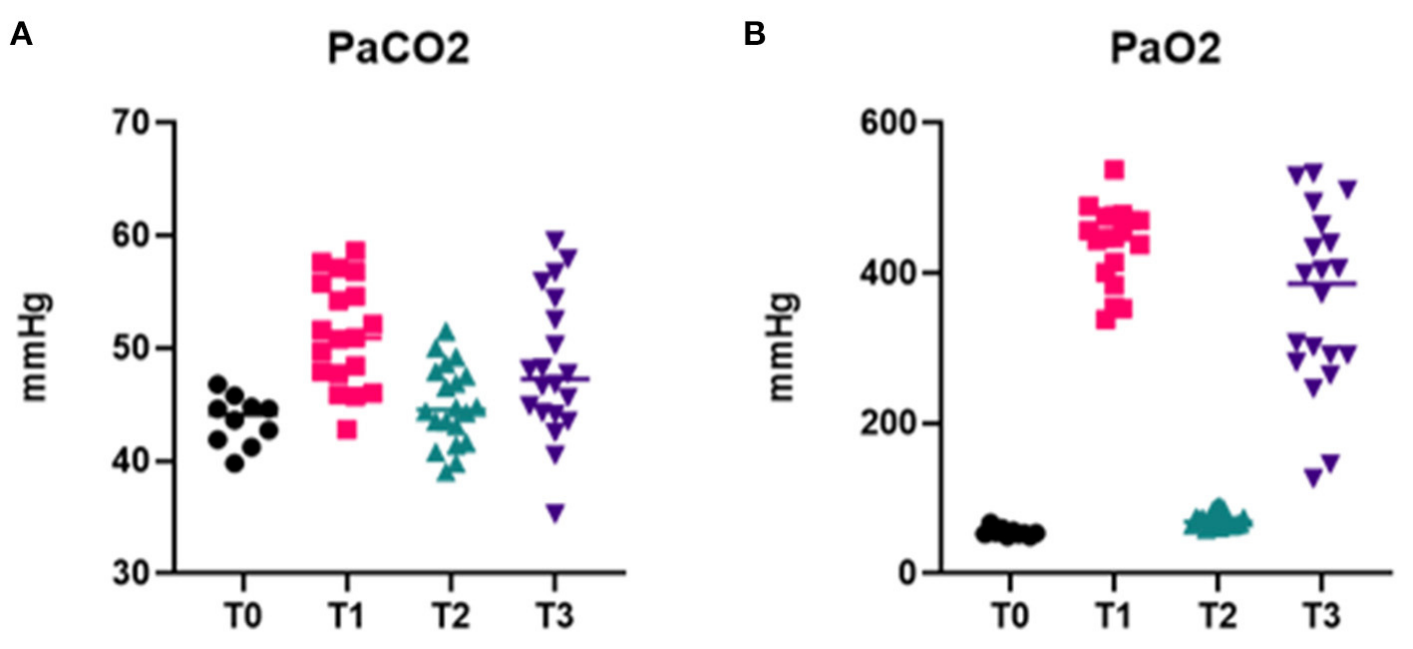
Arterial blood gas data. Graphs showing PaCO_2_ **(A)** and PaO_2_ **(B)** at different time points of the intubation procedure, in presence (T_1_, T_3_) or absence (T_0_, T_2_) of O_2_ supplementation.

Arterial blood gas results are reported in Table 3 and Figure 6. Data analysis showed significantly lower PaO_2_ in absence of oxygen supplementation, at T_0_ (after premedication and pre O_2_ administration) and T_2_ (immediately after intubation), while PaCO_2_ was not significantly different between time points and moderately elevated (Figure 6). No significant differences were observed for different intubation methods. FiO_2_ measured was between 20 and 21% at T_0_ and T_2_ (20.89 ± 0.33 at T_0_ and 20.95 ± 0.68 at T_2_), 97–100% at T_1_ (99.45 ± 0.83), and 98–100% at T_3_ (99.40 ± 0.68).

**Table 3:** Other arterial blood gas parameters at different time points, in presence (T_1_, T_3_) or absence (T_0_, T_2_) of O_2_ supplementation, over the course of the experiment.

**ABG parameters**	**T_**0**_**	**T_**1**_**	**T_**2**_**	**T_**3**_**
pH	7.39 ± 0.06	7.37 ± 0.04	7.42 ± 0.03	7.4 ± 0.04
BE (mmol/L)	0.93 ± 4.47	2.7 ± 3.41	3.83 ± 2.78	4.4 ± 3
HCO_3_ (mmol/L)	26.11 ± 3.94	28.75 ± 3.29	28.73 ± 2.84	29.8 ± 3.21
SO_2_ (%)	83.73 ± 3.07	99.88 ± 0.04	92.32 ± 3.05	99.72 ± 0.38

*Data are presented as mean and standard deviation*.

## Discussion

The results of this study illustrate that v-gel^®^ guided ETI techniques are an effective and technically easy alternative to the traditionally used blind ETI method in large healthy rabbits. V-gel^®^ guided ETI methods demonstrated a significant higher success and were completed in a shorter amount of time when compared to the traditional blind technique, supporting our hypothesis. *Method 1* allowed for the smooth insertion of a cuffed ETT over a polypropylene guide catheter. The use of a polypropylene catheter as a guide for ETT insertion has been previously described by Thompson et al. in a study from 2017 (5). In this study, the guide was inserted into the trachea by direct laryngoscopy. A significant disadvantage of this technique is that it requires the use of a laryngoscope blade, which can be difficult in smaller breeds or juvenile rabbits due to anatomic size restrictions. Moreover, improper insertion of the laryngoscope can inadvertently injure the larynx, tongue, or teeth (5). Using the v-gel^®^ to insert a guide eliminates the risk associated with direct laryngoscopy. In addition, given the availability of the v-gel^®^ in different sizes, the v-gel^®^ guided technique has the potential for use in small breeds and juvenile rabbits. Furthermore, oxygen supplementation can be provided through the v-gel^®^ during the course of the intubation procedure. In this study, the polypropylene catheter used as a guide was smaller (3.5 French) than the one used in the study by Thompson et al. (5 French). Despite the reduced stiffness of the smaller catheter, it was still utilized effectively as a guide, further decreasing the risk of trauma to the larynx and trachea specifically at the tracheal bifurcation.

The laryngoscopic technique has been reported as easy to teach, with a trainee typically achieving success within 1–3 attempts (5). However, the duration of the ETI procedure was not reported. A more recent study from 2019 comparing capnography guided ETI with the laryngoscopic technique reports prolonged intubation times (>540 s) and supplementary sevoflurane administration necessary to regain anesthetic depth appropriate for intubation (16). In the present study, the investigator performing the intubations (AF) did not have previous experience with v-gel^®^ placement. Intubation was successfully achieved in 9 out 10 times, with only one rabbit requiring 2 attempts because of suspected laryngospasm. This finding suggests that this technique has the potential to be easily taught, while requiring a minimal number of attempts to be successful, even when performed by inexperienced personnel. Further studies involving different operators performing the procedure would be necessary to assess the learning curve for v-gel^®^ supported techniques.

While the laryngoscopic technique can be performed by one single operator (5), the presence of an assistant is critical for performing ETI with *Method 1*. After correct v-gel^®^ placement, it is of the utmost importance to maintain the patient's head steady to ascertain correct positioning of the v-gel^®^ over the laryngeal opening. This maneuver can be further supported by ascertaining a normal waveform on the capnography.

Our investigations using an uncuffed tube inserted directly through the v-gel^®^ airway channel using *Method 2* resulted in the shortest intubation duration (1 ± 0.3 min), and only one intubation attempt was necessary to achieve ETI in all the rabbits (see Table 2). The main limitation of this technique is that due to the diameter restrictions of the v-gel^®^ airway channel, the ETT diameter was smaller (2.5 mm), and the tube was uncuffed. The use of a smaller ETT can result in increased airway resistance and increased risk of obstruction due to mucus (5). The system leak was higher (445 ± 59.86 ml) in rabbits intubated with uncuffed ETT and v-gel^®^ using *Method 2* than rabbits intubated with cuffed ETT with *Method 1* (300 ± 100 ml). Furthermore, the leak measured was higher when v-gel^®^ and ETT were in place compared to v-gel^®^ alone (366.7 ± 81.6). In the authors' opinion, the presence of the ETT may partially displace the v-gel^®^ at the larynx and affect the seal created by the v-gel^®^. It is also possible that the performance of this system could be improved by using a larger v-gel^®^ that would allow for the use of a larger diameter ETT and establish a better endotracheal seal. The size of v-gel^®^ used for the experiments described in this study can be considered relatively small considering the manufacturer recommendations. The weight of the rabbits enrolled in this study ranged between 3.5 and 5.1 kg (4.3 ± 0.4) and 4 kg represents the higher end of weight for which a v-gel^®^ size R4 is recommended. Wenger et al. evaluated four different v-gel^®^ channel sizes in higher weight (5.1 ± 0.05 kg) New Zealand White rabbits. Notwithstanding the manufacturer's endorsement for use of a v-gel^®^ size R6 in rabbits weighing >4.5 kg., the R6 was observed to be too large, causing upper airway obstruction; even the next smaller size (R5), partially compressed the larynx in one third of the rabbits (19). The purpose of the present study was to evaluate the v-gel^®^ as a mean to introduce a guide for the ETT. Therefore, we chose a slightly smaller v-gel^®^ size to facilitate device placement to limit trauma to the oropharynx and laryngeal structures as well as to minimize the risk of partial laryngeal compression and obstruction.

The results of this study also confirm the challenges of the traditional blind intubation technique for rabbits. Overall success with blind intubation techniques was lower than for the v-gel^®^-guided techniques. Blind intubation with cuffed ETT (*Method 4*) was successful in only one rabbit, while the correspondent procedure with an uncuffed tube in *Method 3*, was successful in 8 out 10 animals. These findings suggest a potential association of the cuff with increased risk of failure during intubation. This could be explained by the presence of the cuff altering the conformation of the distal end of the ETT, subsequently increasing the difficulty to pass through the larynx. However, it is interesting that in this study, the only animal for which the blind ETI with cuffed ETT was successfully achieved, was also the smallest one in the study group (weight 3.5 kg). Furthermore, the same size cuffed ETT was easily inserted using a stylet in *Method 1*. Therefore, the effect conferred by the cuff itself is not a sufficient explanation for this difference. These results should thus be interpreted cautiously, and further studies are warranted to specifically explore the relationship between ETT design and intubation technique. Since the development and conduction of this study, the v-gel^®^ ADVANCED Rabbit (Docsinnovent) was introduced into the veterinary market. This device is intended for single use, and some of the differences with the original model include modifications in the shape of the laryngeal seal and an increase in the length of the airway channel. At the time of this study, the authors were not aware of the development and availability of this product on the market. The model used in this study, albeit still in clinical use, is no longer available by the manufacturer. The v-gel^®^ ADVANCED Rabbit has begun to be rolled out into clinical practice. To the author's knowledge, a similar study as the one presented herein has not yet been published.

Endoscopic and histological assessment of intubation techniques for rabbits has been performed using the v-gel^®^ and endotracheal tubes (3, 8, 11). In this study, gross macroscopic findings indicated mild to moderate upper airway trauma. We acknowledge the limited utility of upper airway endoscopy findings in our study without supporting histology. Because of the crossover design in this study, lesions or trauma observed prior to intubation attempts could be attributable to damage from a previous anesthetic event. Furthermore, due to the limited availability of images found to be of sufficient quality to make an assessment, upper airway lesions or trauma may have been underestimated. Finally, sacrifice at the conclusion of this study for histologic assessment of laryngeal and tracheal tissue was not performed in this study as rabbits were destined for a long-term survival study. This last limitation is noteworthy as gross endoscopic evaluation of the larynx has shown to underestimate laryngeal trauma secondary to ETI when compared with histology (11).

The arterial blood gas results from our investigations demonstrated the development of a profound hypoxemia in the absence of oxygen supplementation during ETI. Similar findings have been reported for rabbits who were administered a range of anesthetic combinations including α_2_-agonists (21) and are believed to be due to hypoventilation associated with anesthesia. To the authors' knowledge, this is the first report documenting profound hypoxemia occurring during the intubation procedure. The rapid response to oxygen supplementation, demonstrated by increased PaO_2_ values with 5 min of pre-oxygenation preceding intubation, is followed by a dramatic and rapid return to severe hypoxemia when oxygen supplementation is discontinued to perform intubation. Moreover, this response is consistent across different types of intubations and therefore, does not show a correlation with the duration of the procedure. Even the quickest intubation, and thus the shortest time of disruption in oxygen support, resulted in a profound hypoxemia. These findings further support the importance of providing O_2_ supplementation to be critical for safe ETI in the rabbit to minimize the risk of hypoxemia-associated complications. An additional advantage of the v-gel^®^ guided techniques is that this method permits continued oxygen supplementation during the intubation process. This may be particularly advantageous for juvenile or critically ill rabbits, who may be already sensitized toward negative cardiopulmonary consequences following hypoxemia. Increased PaCO_2_ was observed at all time points, with higher values in presence of oxygen supplementation (Figure 6A). However, no statistically significant differences between time points or intubation techniques were found. The hypercapnia can be explained by the pronounced hypoventilation associated with the respiratory depression induced by the combination of ketamine and xylazine (22, 23) and the fact that the rabbits were not mechanically ventilated for the duration of the experiment.

Given that SpO_2_ values recorded in presence or absence of oxygen supplementation were in general above 90%, the arterial blood gas results presented in this study also support the use of blood gas analysis for the monitoring of accurate gas tensions of rabbits under anesthesia. This conclusion has also been supported previously (24, 25). Due to the general ease of auricular arterial catheterization in rabbits and the accuracy of blood gas analysis relative to less invasive techniques (SpO_2_ and ETCO_2_), blood gas analysis should be used to supplement non-invasive monitoring whenever possible due to the respiratory and ventilatory concerns for anesthetized rabbits.

Although ETCO_2_ data were not included in the multivariate analysis, however values recorded in the animals intubated with uncuffed ETT (*Methods 2* and *3*) were lower (Figure 5). In these animals, the capnography measurement was likely inaccurate due to the increased system leak. In the animals intubated with *Method 2*, the presence of the v-gel^®^ connector and the ETT connector resulted in an increase of dead space, which was combined with an increased system leak. In the animals intubated with *Method 3*, the capnography ETCO_2_ reading was inaccurate because of the considerable system leak (>1 L).

In conclusion, we have demonstrated that a v-gel^®^ designed for rabbits can be used to facilitate endotracheal intubation. The v-gel^®^ techniques have the advantages of reducing the number of intubation attempts, of decreasing the overall time of intubation, and of subsequently preventing some of the risks associated with general anesthesia in the rabbit. Specifically, the v-gel^®^ ETI techniques also afford a critical advantage by allowing uninterrupted oxygen supplementation during intubation. We have documented the pronounced hypoxemia that occurs during the peri-intubation process, and the v-gel^®^ techniques allow for the provision of oxygen during the intubation process (e.g., after the v-gel^®^ has been placed but before placement of the polypropylene guide catheter). In addition, v-gel^®^ techniques may potentially decrease laryngeal trauma, which is often caused by multiple repeated blind intubation attempts or by the introduction of a laryngoscope. V-gel^®^-guided techniques allow successful, rapid ETI to provide airway security and ventilatory support for rabbits under general anesthesia. By demonstrating a superior endotracheal intubation technique in rabbits, the morbidity and mortality associated with general anesthesia in rabbits can hereby be improved.

## Data Availability Statement

The raw data supporting the conclusions of this article will be made available by the authors, without undue reservation.

## Ethics Statement

The animal study was reviewed and approved by Institutional Animals Care and Use Committee, University of Pennsylvania.

## Author Contributions

AF, KH, HD, and TS: study design. AF, KH, HD, and AB: data collection. DS, AF, and HD: data analysis and interpretation. All authors manuscript drafting and final approval.

## Conflict of Interest

The authors declare that the research was conducted in the absence of any commercial or financial relationships that could be construed as a potential conflict of interest.

## Publisher's Note

All claims expressed in this article are solely those of the authors and do not necessarily represent those of their affiliated organizations, or those of the publisher, the editors and the reviewers. Any product that may be evaluated in this article, or claim that may be made by its manufacturer, is not guaranteed or endorsed by the publisher.
