# Supplementary material for: V-Gel® Guided Endotracheal Intubation in Rabbits
Source: Front Vet Sci. 2021 Aug 10;8:684624. doi: 10.3389/fvets.2021.684624 (PMC8383107; doi:10.3389/fvets.2021.684624)
Supplement: Supplementary file 1 [file Data_Sheet_1.pdf]

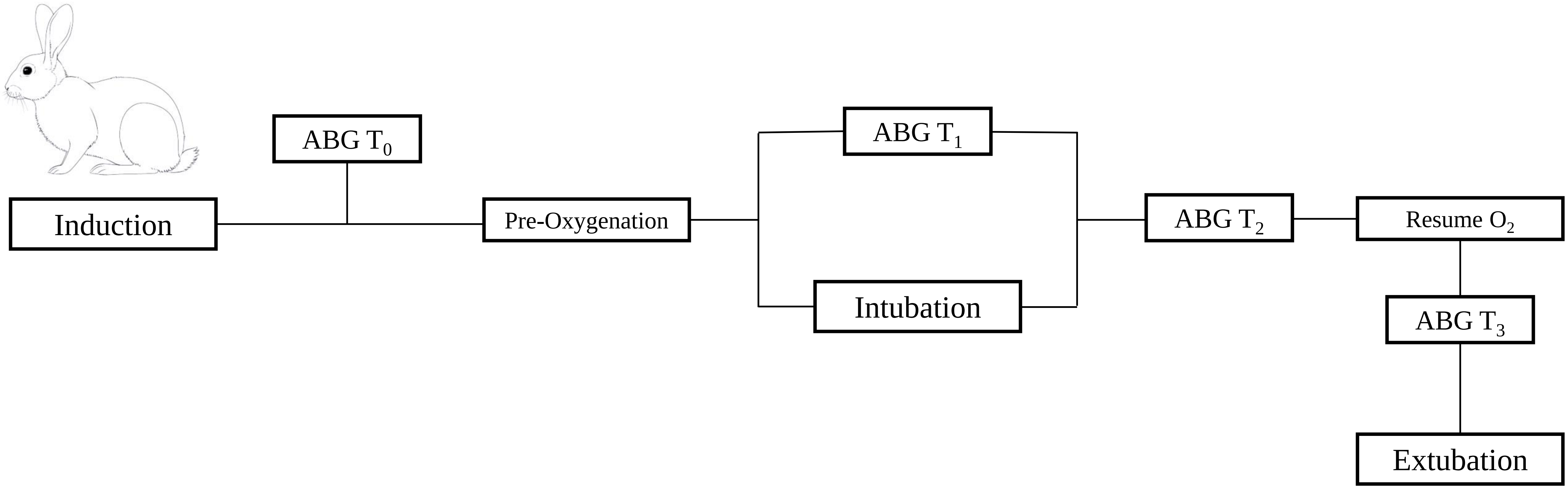

Diagram showing the timeline of events for the intubation experiments.
ABG: Arterial blood Gas

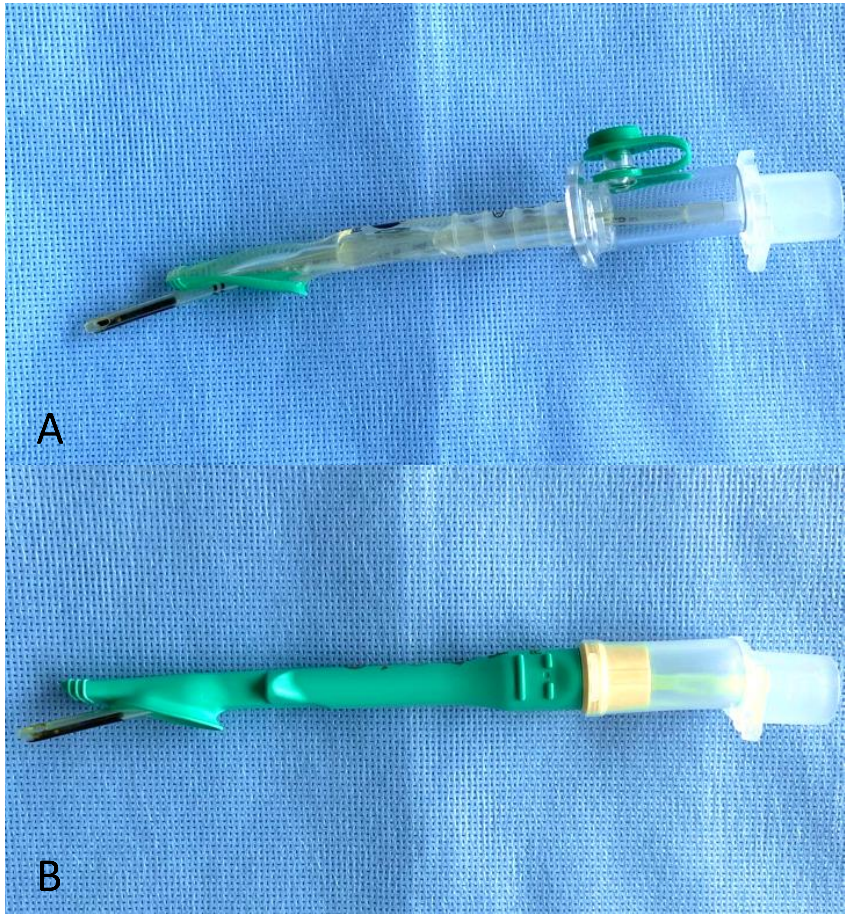

Figure 1: Photographs showing a 2.5 internal diameter endotracheal tube inserted through the airway channel of the old design v-gel® (A) and the new v-gel® ADVANCED Rabbit (B).
